# Assessment of thyroid disorders in patients with rosacea: a large case-control study^☆^^☆☆^

**DOI:** 10.1016/j.abd.2021.02.004

**Published:** 2021-07-16

**Authors:** Asli Akin Belli, Emine Tugba Alatas, Asude Kara Polat, Gulhan Akbaba

**Affiliations:** a Mugla Sitki Kocman University Training and Research Hospital, Department of Dermatology, Mugla, Turkey; b Mugla Sitki Kocman University Medical School, Department of Dermatology, Mugla, Turkey; c Istanbul Training and Research Hospital, Department of Dermatology, Istanbul, Turkey; d Mugla Sitki Kocman University Medical School, Department of Endocrinology and Metabolism Diseases, Mugla, Turkey

**Keywords:** Hypothyroidism, Rosacea, Thyroid diseases

## Abstract

**Background:**

The frequency of autoimmune diseases and thyroid cancer has been increasingly reported in association with rosacea. However, studies investigating thyroid diseases in rosacea are scarce with conflicting results.

**Objective:**

To investigate the relationship between thyroid disorders and rosacea.

**Methods:**

A large case-control study on age- and gender-matched 2091 rosacea patients and 9572 controls was conducted. Rosacea patients using the rosacea-specific ICD codes were compiled from the hospital records. Additionally, all participants were evaluated in terms of the presence of hypothyroidism and hyperthyroidism. Conditional logistic regression analysis was used to compute case-control odds ratios (OR) with 95% confidence intervals.

**Results:**

The analysis comprehended 2091 rosacea patients (1546 female, 545 male; mean 48.73 ± 14.53 years) and 9572 controls (7009 female, 2563 male; mean 48.73 ± 15.1 years). Whereas the rate of hypothyroidism was significantly higher in rosacea patients (OR = 1.3, 95% CI 1.13–1.49, p < 0.001), there was no significant difference in the rate of hyperthyroidism between the groups (OR = 1.12, 95% CI 0.81–1.53, p = 0.497). Stratification for gender revealed a significant association between hypothyroidism and rosacea in females (OR = 1.27, 95% CI 1.1–1.47, p = 0.002) and males (OR = 1.58, 95% CI 1.04–2.4, p = 0.032). The frequency of hypothyroidism in rosacea patients increased towards the age range of 40–49 and then decreased, parallel with the hypothyroidism frequency of the study population.

**Study limitations:**

Different subtypes and severities of rosacea were not distinguished.

**Conclusions:**

Hypothyroidism may be a comorbidity of rosacea and investigation for hypothyroidism may be appropriate when evaluating rosacea patients.

## Introduction

Rosacea is a chronic inflammatory cutaneous disease that can be characterized by recurrent episodes of erythema, papules, pustules, phymatous changes, and ocular symptoms. Rosacea is more frequent particularly in fair-skinned populations with a prevalence range of 1–10 percent.^1^, ^2^ Although various factors including immune dysregulation, vascular hyperreactivity, microorganisms, Ultraviolet radiation (UV), and more rarely genetics have been proposed in the pathogenesis, it has not been fully understood.^2^, ^3^ Moreover, diverse comorbidities such as cardiovascular, gastrointestinal, neurologic, and psychiatric diseases have been found to increase in rosacea patients.^4^, ^5^

In recent years, the relationship between rosacea and autoimmune diseases involving type 1 diabetes mellitus, celiac disease, rheumatoid arthritis, and multiple sclerosis has been emphasized.^6^, ^7^ However, whereas the frequency of thyroid cancer has also been reported to increase in rosacea patients, thyroid autoimmunity has not been studied deeply in rosacea.^8^ The relation between thyroid autoimmunity and thyroid cancer has been known for a long time.^9^ Furthermore, similar to thyroid disorders, the association between rosacea and metabolic disorders has been reported in detail in recent years.^10^, ^11^, ^12^, ^13^

The authors hypothesized that shared pathogenic mechanisms can provide a link between rosacea and thyroid disorders and thus, the authors aimed to investigate whether there is a relationship between thyroid disorders (hypo- and hyperthyroidism) and rosacea.

## Methods

A large case-control study on 2091 rosacea patients and 9572 age- and gender-matched controls who were admitted to the Dermatology Outpatient Clinic of Mugla Sitki Kocman University Training and Research Hospital between January 1, 2015, and December 31, 2019, was conducted. Ethics committee approval was obtained from the Mugla Sitki Kocman University Ethics Committee. Rosacea patients were identified using ICD (International Classification of Diseases) codes of rosacea (L71.8 and L71.9) and were compiled from records of the hospital. Controls were selected consecutively from the patients who were admitted to the study’s outpatient clinic with various dermatological complaints except for rosacea. Each rosacea patient was matched with approximately five age- and gender-matched controls (on calendar time). All participants were evaluated for the presence of hypothyroidism and hyperthyroidism by using disease-specific ICD codes (E03.8 and E03.9; E05.9 and E0.2, respectively). The inclusion criteria of the study were being of ≥18 years for the patients and controls.

The SPSS for windows 22.0 software was employed for the statistical analysis. The descriptive statistics were demonstrated as mean, standard deviation, and frequency. The distribution of variables was checked with the Kolmogorov-Smirnov test. Because all of the numeric variables were not distributed normally, Mann-Whitney *U*-test was used for the quantitative data. The Chi-Square test was used for the analysis of qualitative data. Conditional logistic regression analysis was used to compute case-control Odds Ratios (OR) with 95% Confidence Intervals; p-value <0.05 was assessed as significant.

## Results

2091 rosacea patients (1546 female, 545 male; age range 18–89 years, mean 48.73 ± 14.53) and 9572 controls (7009 female, 2563 male; age range 18–89 years, mean 48.73 ± 15.1) were matched on age, gender, and calendar time in the study (Table 1).

**Table 1:** Characteristics of the study population.

	Rosacea group (n = 2091)	Control group (n = 9572)	p
**Age (years)(mean ± SD)**	48.73 ± 14.53	48.73 ± 15.1	0.942
**Gendern (%)**			
Female	1546 (73.9)	7009 (73.2)	0.505
Male	545 (26.1)	2563 (26.8)	

Chi-square test, Mann Whitney *U* test. SD, Standard Deviation.

Whereas the prevalence rate of hypothyroidism was significantly higher in rosacea patients (OR = 1.3, 95% CI 1.13–1.49, p < 0.001), there was no significant difference in the rate of hyperthyroidism between rosacea patients and controls (OR = 1.12, 95% CI 0.81–1.53, p = 0.497) (Table 2).

**Table 2:** Odds ratios linking rosacea with the occurrence of hypothyroidism and hyperthyroidism.

	Rosacea group n (%)	Control group n (%)	OR	95% CI	p
**Hypothyroidism**					
Present	300 (14.3)	1083 (11.3)	1.3	1.13–1.49	<0.001
Absent	1791 (85.7)	8489 (88.7)			
**Hyperthyroidism**					
Present	49 (2.3)	204 (2.1)	1.12	0.81-1.53	0.497
Absent	2042 (97.7)	9368 (97.9)			

Conditional logistic regression. OR, Odds Ratio; CI, Confidence Interval.

Stratification for gender also revealed a significant association between hypothyroidism and rosacea in females (OR = 1.27, 95% CI 1.1–1.47, p = 0.002) and males (OR = 1.58, 95% CI 1.04–2.4, p = 0.032, respectively), whereas there was no significant association between hyperthyroidism and rosacea in both genders (p < 0.05) (Table 3).

**Table 3:** Odds ratios linking rosacea with the occurrence of hypothyroidism and hyperthyroidism stratified by gender.

	n (%)	OR	95% CI	p
**Hypothyroidism**				
Female	269 (17.4)	1.27	1.1 – 1.47	0.002
Male	31 (5.7)	1.58	1.04 – 2.4	0.032
**Hyperthyroidism**				
Female	37 (2.4)	1.1	0.77 – 1.58	0.618
Male	12 (2.2)	1.18	0.62 – 2.22	0.617

Conditional logistic regression. OR, Odds Ratio; CI, Confidence Interval.

The frequency of hypothyroidism in rosacea patients increased towards the age range of 40–49 and then decreased towards the age range of 80–89, as compatible with the general hypothyroidism frequency of the study population (Table 4).

**Table 4:** Frequency of hypothyroidism in the participants by ~10-year age intervals.

	Rosacea patients with hypothyroidism	Controls with hypothyroidism	All participants with hypothyroidism
Age (years)	n (%)	n (%)	n (%)
18 to 29	14 (0.7)	68 (0.7)	82 (0.7)
30 to 39	63 (3)	226 (2.4)	289 (2.5)
40 to 49	94 (4.5)	284 (3)	378 (3.2)
50 to 59	77 (3.7)	281 (2.9)	358 (3.1)
60 to 69	37 (1.8)	157 (1.6)	194 (1.7)
70 to 79	14 (0.7)	61 (0.6)	75 (0.6)
80 to 89	1 (0.1)	6 (0.1)	7 (0.1)

## Discussion

Rosacea is a chronic and inflammatorydisease whose pathogenesis has been still investigated. Moreover, a growing number of comorbidities are being reported in rosacea, suggesting physicians evaluate rosacea patients more deeply.^4^, ^5^ Factors that encouraged us to conduct the present study comprised (i) rosacea has been associated with some autoimmune diseases, (ii)thyroid cancer has been reported to increase in rosacea patients, and (iii) thyroid disorders are also associated with the inflammatory process, as well as cardiovascular conditions.^6^, ^8^, ^10^ In this study, the frequency of hypothyroidism was significantly increased in rosacea patients and no association between hyperthyroidism and rosacea was shown. After stratification for gender, the association between hypothyroidism and rosacea continued in both genders. The age range in which hypothyroidism was most common was 40 to 49 in rosacea patients, compatible with the all study population in terms of hypothyroidism frequency.

In recent years, several studies have been reported suggesting autoimmunity in rosacea. Egeberg et al. reported that rosacea is associated with type 1 diabetes mellitus, multiple sclerosis, celiac disease, and rheumatoid arthritis, particularly in women.^6^ *Helicobacter pylori* infection which has been involved in the pathogenesis of rosacea can be emphasized as another suggestive finding of autoimmunity in rosacea. Indeed, diverse autoimmune diseases such as rheumatoid arthritis, autoimmune thyroiditis, and Henoch-Schoenlein purpura have been associated with *Helicobacter pylori* infection besides rosacea.^14^, ^15^ Moreover, Wozniacka et al. have reported that 53.3% of 101 rosacea patients had an ANA titer ≥ 1:160 and none of the patients with high ANA titers developed a known autoimmune disorder during the two-year follow-up period.^16^ However, although most of thyroid diseases are autoimmune, thyroid diseases have not been studied much in rosacea.^17^

The relation between rosacea and thyroid disorders has been investigated in a few studies, with conflicting results. James et al. reported hypothyroidism in 17.5% of psoriasis and in 19.7% of rosacea patients. Although the rate of hypothyroidism has been noted as relatively low (13.8%) in patients with other dermatologic diseases, they have not mentioned the difference as significant.^18^ In another study, Berksoy Hayta et al. have investigated serum levels of free thyroid hormones, thyroid autoantibodies, prolactin, Dehydroepiandrosterone Sulfate (DHEAS), basal cortisol, C-reactive protein, and erythrocyte sedimentation rates in 72 rosacea patients and 62 controls, and reported no significant association between the groups according to the presence of thyroid disease. However, the levels of CRP, anti-microsomal antibody (anti-M), and prolactin were increased in rosacea patients.^19^ Artantaş et al. reported acne rosacea in 3.6% of 220 patients with thyroid disease^20^ In the present study, the rate of hypothyroidism was significantly higher in the rosacea group (14.3%) than in the control group (11.3%), even in both genders separately. However, since serum thyroid autoantibodies were not evaluated in all participants, laboratorial data confirming autoimmune thyroid disease were not demonstrated.

While comorbidities have been investigating in rosacea, the frequency of thyroid cancer has been evaluated in a few studies. Whereas Li et al. have reported an increased risk of thyroid cancer in rosacea patients in the US, Egeberg et al. have shown no increased risk of thyroid cancer in rosacea patients in Danish population.^8^, ^21^ The inflammatory response and immune function play a pivotal role in the pathogenesis of thyroid cancer.^22^ Indeed, thyroid autoimmunity and thyroid cancer, particularly papillary thyroid cancer, have been studied and associated for several decades.^9^ Inflammation may be one potential link between rosacea and thyroid cancer. The association between rosacea and thyroid cancer reported may support the present study’s results considering that autoimmune hypothyroidism is the most common cause of hypothyroidism, as well as thyroid autoimmunity and thyroid cancer are highly correlated.^9^, ^22^

Rosacea and thyroid diseases are also similar in terms of accompanying metabolic conditions and inflammatory pathways. Thyroid hormones have a key role in lipid and glucose metabolism, energy homeostasis, and also blood pressure. Therefore, a glucose-lipid metabolism disorder, hypercholesterolemia, and atherosclerosis are more frequent in patients with hypothyroidism.^10^ Rosacea has also been associated with cardiovascular diseases in several studies with similar metabolic changes found in hypothyroidism.^11^, ^12^, ^13^ Moreover, increased expression of inflammatory markers including matrix metalloproteinases, particularly MMP-9, has been demonstrated in rosacea as well as in hypothyroidism.^23^, ^24^, ^25^, ^26^

The authors had some limitations in the present study. Firstly, this study was a database study and thus, was prone to coding errors. The authors were unable to distinguish the different subtypes and severities of rosacea, and also thyroid disorders as autoimmune or non-autoimmune disease. Another limitation of the present study was to be conducted in Turkish people and in one center, and thus, the failure of generalization of the results. Additionally, the present study, as an observational study, did not determine causality whether rosacea leads to hypothyroidism or vice versa.

## Conclusion

In conclusion, the rate of hypothyroidism was significantly increased in rosacea patients, even after gender stratification in both genders. The frequency of hypothyroidism was highest in the age range of 40 to 49 in rosacea patients, as compatible with all the participants. Hypothyroidism may be involved among the comorbidities of rosacea and investigation for hypothyroidism may be an appropriate approach when evaluating rosacea patients. Future studies are needed to confirm the association between rosacea and hypothyroidism in other populations, to explain the common pathways of these diseases in detail, and to recommend routine examination for accompanying thyroid disorders in rosacea patients.

## Financial support

None declared.

## Authors' contributions

Asli Akin Belli: Approval of the final version of the manuscript; critical literature review; data collection, analysis and interpretation; manuscript critical review; preparation and writing of the manuscript; statistical analysis; study conception and planning.

Emine Tugba Alatas: Approval of the final version of the manuscript; critical literature review; data collection, analysis and interpretation; manuscript critical review; preparation and writing of the manuscript; statistical analysis; study conception and planning.

Asude Kara Polat: Approval of the final version of the manuscript; data collection, analysis and interpretation; manuscript critical review; preparation and writing of the manuscript.

Gulhan Akbaba: Approval of the final version of the manuscript; critical literature review; data collection, analysis and interpretation; manuscript critical review; study conception and planning.

## Conflicts of interest

None declared.

## References

[1] Elewski B.E., Draelos Z., Dréno B., Jansen T., Layton A., Picardo M. (2011). Rosacea - global diversity and optimized outcome: proposed international consensus from the Rosacea International Expert Group. J Eur Acad Dermatol Venereol..

[2] Marson J.W., Baldwin H.E. (2020). Rosacea: a wholistic review and update from pathogenesis to diagnosis and therapy. Int J Dermatol..

[3] Crawford G.H., Pelle M.T., James W.D. (2018). Rosacea: I. Etiology, pathogenesis, and subtype classification. J Am Acad Dermatol..

[4] Haber R., El Gemayel M. (2018). Comorbidities in rosacea: A systematic review and update. J Am Acad Dermatol..

[5] Holmes A.D., Spoendlin J., Chien A.L., Baldwin H., Chang A.L.S. (2018). Evidence-based update on rosacea comorbidities and their common physiologic pathways. J Am Acad Dermatol..

[6] Egeberg A., Hansen P.R., Gislason G.H., Thyssen J.P. (2016). Clustering of autoimmune diseases in patients with rosacea. J Am Acad Dermatol..

[7] Egeberg A., Weinstock L.B., Thyssen E.P., Gislason G.H., Thyssen J.P. (2017). Rosacea and gastrointestinal disorders: a population-based cohort study. Br J Dermatol..

[8] Li W.Q., Zhang M., Danby F.W., Han J., Qureshi A.A. (2015). Personal history of rosacea and risk of incident cancer among women in the US. Br J Cancer..

[9] Nagayama Y. (2018). Thyroid Autoimmunity and Thyroid Cancer - The Pathogenic Connection: A. 2018 Update. Horm Metab Res..

[10] Lei Y., Yang J., Li H., Zhong H., Wan Q. (2019). Changes in glucose-lipid metabolism, insulin resistance, and inflammatory factors in patients with autoimmune thyroid disease. J Clin Lab Anal..

[11] Hua T.C., Chung P.I., Chen Y.J., Wu L.C., Chen Y.D., Hwang C.Y. (2015). Cardiovascular comorbidities in patients with rosacea: A nationwide case-control study from Taiwan. J Am Acad Dermatol..

[12] Belli A.A., Altun I., Altun I. (2017). Thickness of carotid intima and epicardial fat in rosacea: a cross-sectional study. An Bras Dermatol..

[13] Chen Q., Shi X., Tang Y., Wang B., Xie H.F., Shi W. (2020). Association between Rosacea and Cardiometabolic Disease: A Systematic Review and Meta-Analysis. J Am Acad Dermatol..

[14] Yang X. (2018). Relationship between Helicobacter pylori and Rosacea: review and discussion. BMC Infect Dis..

[15] Gasbarrini A., Franceschi F., Does H. (2005). Pylori infection play a role in idiopathic thrombocytopenic purpura and in other autoimmune diseases?. Am J Gastroenterol..

[16] Woźniacka A., Salamon M., McCauliffe D., Sysa-Jędrzejowska A. (2013). Antinuclear antibodies in rosacea patients. Postepy Dermatol Alergol..

[17] Hollowell J.G., Staehling N.W., Flanders W.D., Hannon W.H., Gunter E.W., Spencer C.A. (2002). Serum TSH, T(4), and thyroid antibodies in the United States population (1988 to 1994): National Health and Nutrition Examination Survey (NHANES III). J Clin Endocrinol Metab..

[18] James S.M., Hill D.E., Feldman S.R. (2016). Hypothyroidism in Patients with Psoriasis or Rosacea: A Large Population Study. Dermatol Online J..

[19] Hayta B.S., Guner R., Cam S., Akyol M. (2018). Rosacea is associated with thyroid autoimmunity: a case control study. Acta Endocrinol (Buchar)..

[20] Artantaş S., Gül U., Kiliç A., Güler S. (2009). Skin findings in thyroid diseases. Eur J Intern Med..

[21] Egeberg A., Fowler J.F., Gislason G.H., Thyssen J.P. (2017). Rosacea and risk of cancer in Denmark. Cancer Epidemiol..

[22] Bozec A., Lassalle S., Hofman V., Ilie M., Santini J., Hofman P. (2010). The thyroid gland: a crossroad in inflammation-induced carcinoma?. An ongoing debate with new therapeutic potential. Curr Med Chem..

[23] Buddenkotte J., Steinhoff M. (2018). Recent advances in understanding and managing rosacea. F1000Res..

[24] Ahn C.S., Huang W.W. (2018). Rosacea Pathogenesis. Dermatol Clin..

[25] Marfella R., Ferraraccio F., Rizzo M.R., Portoghese M., Barbieri M., Basilio C. (2011). Innate immune activity in plaque of patients with untreated and L-thyroxine-treated subclinical hypothyroidism. J Clin Endocrinol Metab..

[26] Jublanc C., Beaudeux J.L., Aubart F., Raphael M., Chadarevian R., Chapman M.J. (2011). Serum levels of adhesion molecules ICAM-1 and VCAM-1 and tissue inhibitor of metalloproteinases, TIMP-1, are elevated in patients with autoimmune thyroid disorders: relevance to vascular inflammation. Nutr Metab Cardiovasc Dis..

